# A larger amount of palatable food is needed to provide stress relief during Western diet-induced obesity

**DOI:** 10.1016/j.physbeh.2026.115226

**Published:** 2026-01-08

**Authors:** Ivanka Rainer, Leah Hershberger, Megan Pedicini, Madison Davis, Yvonne M. Ulrich-Lai

**Affiliations:** a Department of Pharmacology, Physiology, and Neurobiology, University of Cincinnati, Cincinnati, OH, 45237, USA; b Neuroscience Graduate Program, University of Cincinnati, Cincinnati, OH, USA

**Keywords:** Sucrose, Stress, HPA axis, Obesity, Corticosterone, Food reward

## Abstract

Many individuals eat highly palatable foods to cope with stress, and this so-called ‘comfort’ feeding occurs to a greater extent in people who are overweight and obese. To study this relationship, we previously characterized a limited sucrose intake (LSI) paradigm that reduces hypothalamic-pituitary-adrenocortical (HPA) axis stress responses in normal weight rats, but not in those with Western diet-induced obesity (DIO). The present work tests the hypothesis that a larger volume of sucrose drink recovers effective HPA blunting during obesity. Time-limited sucrose access, in which sucrose was offered in an unlimited volume for 30 min twice-daily to chow-fed lean and Western DIO rats for 2 weeks, resulted in greater sucrose drink intake than that allowed in the LSI paradigm (4 ml/twice-daily session) and reduced post-stress plasma corticosterone relative to water controls in both lean and DIO rats. Likewise, when given volume-limited access to a larger sucrose volume (6 ml vs. the standard 4 ml per twice-daily session) the 6 ml volume reduced post-stress plasma corticosterone relative to water controls, in both lean and DIO rats, whereas the 4 ml volume was only effective in lean rats. These data replicate that the typical LSI sucrose volume does not produce effective stress-blunting during Western DIO, and extend this to show that larger sucrose volumes, given via either time- or volume-limited access paradigms, recovers this effect. This suggests that stress-related eaters with obesity may require larger amounts of palatable foods to maintain adequate stress relief.

## 1. Introduction

Obesity is a common metabolic disorder that is associated with several comorbidities, including increased risk of stress-related disorders like hypertension, heart disease, depression, and anxiety [1–4]. Individuals with obesity self-report higher levels of stress and are more likely to engage in “comfort” feeding – the consumption of highly palatable, often calorically dense, food to blunt the negative effects of stress [5–7]. Consuming highly palatable foods prior to or following a stressful event decreases perceived and physiological stress levels, though this stress relief comes at the cost of increased risk for developing obesity or overweight [8–10]. These findings illustrate the complex interactions between stress-related palatable feeding and metabolic health, underscoring the importance of understanding how the consumption of highly palatable foods reduces stress, as well as how this process is impacted by obesity.

Our lab established a limited sucrose intake (LSI) paradigm in rats to investigate the mechanisms by which palatable food intake blunts stress responses [11–14]. This paradigm provides palatable food as a choice, in a limited amount, and on an intermittent schedule, thereby maximizing its rewarding properties and minimizing its metabolic (i.e., obesogenic) impact. During LSI, rats with free access to their normal chow and water are given additional twice-daily access to a small amount of sucrose drink (4 mL of 30 % sucrose solution vs water control) for two weeks. This small amount of sucrose provides approximately 10 % of daily caloric intake and does not alter body weight or adiposity, as the rats readily decrease their chow intake to compensate for the sucrose calories [12,14]. Importantly, prior LSI reduces the plasma corticosterone responses to an acute psychogenic (restraint) stressor [12,15,16], indicating effective blunting of the hypothalamic-pituitary-adrenocortical (HPA) axis response to stress. This LSI-induced HPA blunting is prevented by direct placement of the sucrose into the stomach (thereby minimizing the pleasurable taste of the sucrose), and is replicated by a history of limited access to a non-caloric sweetener (0.1 % saccharin drink), another type of palatable food (cheese), and another type of natural reward (sexual activity) [17,18], suggesting that the stress blunting is primarily due to the hedonic and rewarding properties of sucrose.

Despite clear evidence that LSI effectively reduces HPA axis stress responses in normal weight rodents, this effect does not occur in rats with Western diet-induced obesity (DIO) [11]. This may be due to a state of consummatory reward deficit, as both people with obesity and DIO rats have decreased reward system activation in response to palatable food consumption [19,20]. This suggests the possibility that the amount of consummatory food reward provided by LSI is insufficient to confer stress relief during obesity, and therefore greater amounts of sucrose intake may be needed to produce effective HPA axis blunting. The present study uses two different behavioral approaches (time-limited and volume-limited sucrose drink access) to increase sucrose consumption and compares effects in lean vs. DIO rats to test the hypothesis that greater amounts of sucrose intake recover effective HPA axis-blunting in the context of obesity.

## 2. Methods

### 2.1. Animals

**Fig. 1.**
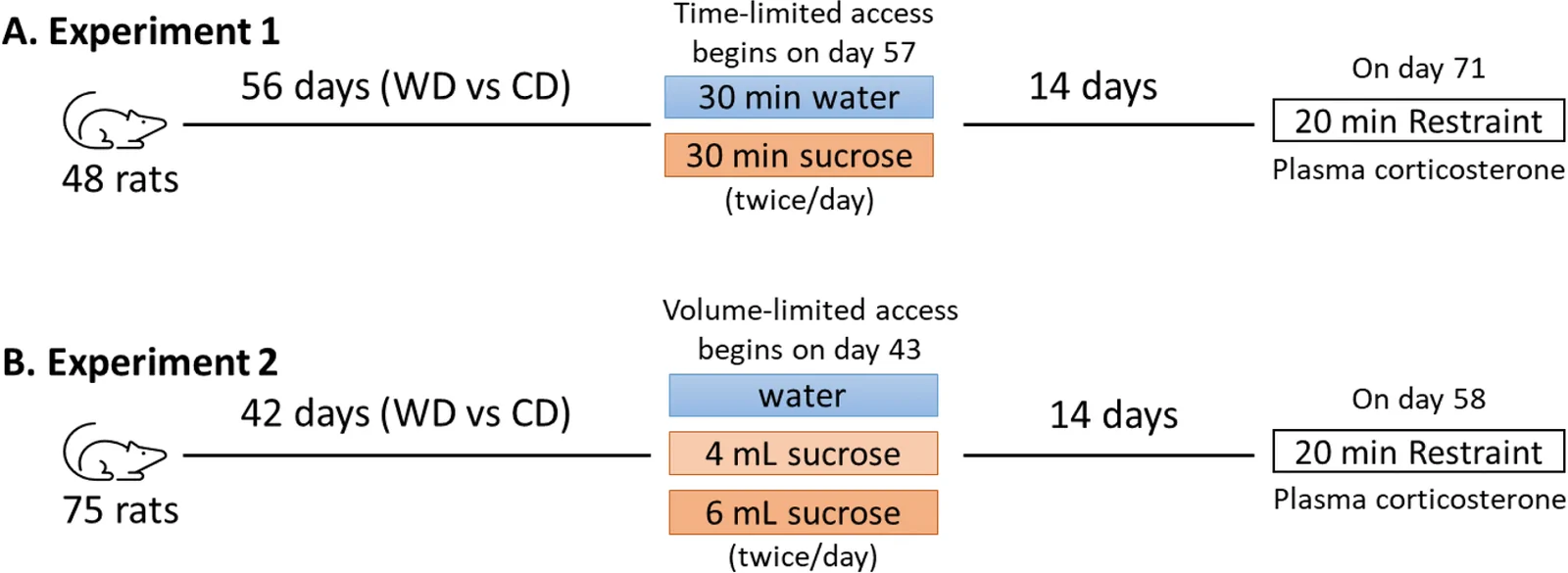
Schematic representation of the experiment timelines. (A) In experiment 1, diet treatment with either Western diet (WD) or chow diet (CD) began on day 1 and continued throughout the remainder of the experiment. Twice-daily time-limited sucrose access began on day 57 and continued for 14 days. On day 71, rats underwent a 20-minute restraint stress for measurement of the plasma corticosterone. (B) In experiment 2, diet treatment began on day 1 and continued throughout the remainder of the experiment. Twice-daily volume-limited sucrose access began on day 43 and continued for 14 days. On day 58, rats underwent a 20-minute restraint stress for plasma corticosterone assessment. Not shown on figure, pre-diet and post-diet NMR measurements of body composition occurred on experiment days 1 and 56 for experiment 1, and on days 1 and 42 for experiment 2.

Adult male Long-Evans rats, weighing approximately 250–275 g, were purchased from Envigo (Indianapolis, IN). Rats were single housed in a temperature- and humidity-controlled housing facility on a 12/12-hour light cycle (lights on at 06:00 h, lights off at 18:00 h) with ad libitum access to food and water. The rodent housing facilities were accredited by the Association for the Assessment and Accreditation of Laboratory Animal Care (AAALAC). Animals accommodated to the animal facilities for at least one week before the experiments started. All procedures were approved by the University of Cincinnati Institutional Animal Care and Use Committee and are consistent with the United States’ National Institutes of Health (NIH) “Guide for the Care and Use of Laboratory Animals”[21]. Rats were divided into two dietary groups that were matched for starting body weight and percent body fat to minimize any pre-existing groups differences that could confound interpretation of dietary effects. To produce DIO, one group of rats was given ad libitum access to a Western diet (WD; D12492, Research Diet, NJ, USA), providing 60 % daily calories from fat, 20 % from protein, and 20 % from carbohydrate, with 35 % of the carbohydrates coming from sucrose, starting 8 weeks prior (experiment 1) or 6 weeks prior (experiment 2) to LSI exposure (Fig. 1). Lean control rats were instead maintained on normal chow diet (CD; Teklad LM-485, product #7012, Envigo, Madison. WI, USA; 17 % daily calories from fat, 25 % from protein, and 58 % from carbohydrate with no added sucrose). Body weight and food intake were monitored to confirm that WD effectively increased caloric intake and body weight. Pre- (measured on experiment day 1) and post-diet (measured on experiment day 56 (experiment 1) or day 42 (experiment 2)) body composition was measured by nuclear magnetic resonance (EchoMRI, Houston, TX) imaging to confirm that WD effectively increased adiposity. We have previously shown that both 6- and 8-weeks of high-fat diet exposure reliably induces obesity and metabolic dysfunction, resulting in increased body weight and body fat, impaired glucose tolerance, insulin insensitivity, and increased plasma leptin levels [11,22–24].

### 2.2. Sucrose intake paradigms

In the typical LSI paradigm, rats with free access to their normal water bottle and their assigned diet are given additional twice-daily access (at 09:00 h and 16:00 h) to up to 4 ml of 30 % sucrose drink vs. water (as a control) for 2 weeks. The LSI drink session for each rat that is offered 30 % sucrose ends when the rat consumes the full 4 ml of drink, or at 30 min, whichever comes first. The duration of the LSI drink session for the water control rats is matched to the duration of the sucrose group (as rats offered water do not consume the full 4 ml). Typically, rats offered sucrose begin to drink the solution immediately after its placement on their cage and rapidly drink the full 4 ml within about 5 min, indicating that they would likely drink a much larger sucrose volume if it was allowed, thereby providing the opportunity to test the effects of increased sucrose drink intake. This was accomplished via two complimentary behavioral approaches – time-limited sucrose access and volume-limited sucrose access.

#### 2.2.1. Experiment 1: Time-limited access

This procedure was identical to the typical LSI paradigm except that rats were given twice daily access to an *unlimited* amount of 30 % sucrose solution (vs. water as a control) for a *set duration* of time (30 min) and the volume of consumed was recorded. Thus, rats were allowed to drink as much sucrose as they wanted within each 30-min session, and this occurred twice daily at 09:00 h and 14:00 h for two weeks.

#### 2.2.2. Experiment 2: Volume-limited intake

This procedure was identical to the typical LSI paradigm except that a larger maximum volume of 30 % sucrose drink (6 ml) was offered twice daily for two weeks, and effects were compared to the standard sucrose drink volume (4 ml), or water (4 ml) as a control. Thus, the LSI drink session for each rat that was offered either 6 ml or 4 ml of 30 % sucrose ended when the rat consumed the full volume of drink, or at 30 min, whichever came first. The duration of the LSI drink session for the water control rats was matched to the duration of the 6 ml sucrose group (as rats offered water do not consume the full volume offered).

### 2.3. Plasma corticosterone response to an acute restraint stressor

On the morning following the final sucrose drink exposure, rats did not receive their respective LSI drink. Rats were instead given a novel 20-minute restraint stress (beginning at 08:00 h and ending by 12:00 h) to evaluate how a history of LSI alters the HPA axis stress response in the presence or absence of Western DIO. Rats were individually transported in their home cage to the procedure room and quickly placed into a clear, ventilated, plastic restraint device. Tail clip blood samples (200 μl each) were collected into chilled EDTA-coated tubes at 0, 20, 40, and 60 min timepoints after the onset of restraint. Blood collection was completed within 3 min of first touching each rat’s cage, thereby ensuring that the 0-min sample reflected basal, non-stress plasma corticosterone levels [25]. Blood samples were centrifuged (3000 g, 4 °C, 15 min), and plasma was collected for measurement of corticosterone concentrations by radioimmunoassay (RIA) (Corticosterone Double Antibody I125 RIA Kit, MP Biomedicals, Solon, OH).

### 2.4. Statistical analysis

Data are shown as mean ± SEM. For comparing multiple groups, data were analyzed by ANOVA, with repeated measures when appropriate (RM-ANOVA). In addition, *a priori* planned two-tailed *t*-test comparisons were performed to compare sucrose to water within a given diet and time point, and WD to normal chow diet within a drink type and time point. Potential outliers were evaluated as in our prior work [11,12, 14,15,18] using two standard outlier tests: 1) outliers were values that were more than 1.96 times the standard deviation from the mean, and 2) outliers were values that were below the lower quartile or above the upper quartile by more than 1.5 times the interquartile range. Individual data points were removed only if they failed both tests. The integrated plasma corticosterone response was calculated as the area under the curve (AUC) of the time course data using the trapezoidal rule. Statistical significance was set as *p* < 0.05. All significant main and interactive effects are reported in the results section with accompanying F-statements and p-values; non-significant main or interactive effects are not mentioned. Data analysis was performed using GraphPad Prism (La Jolla, CA, USA).

## 3. Results

### 3.1. Experiment 1: Time-limited sucrose

#### 3.1.1. Pre-exposure to WD effectively increased caloric intake, body weight, and adiposity

**Fig. 2.**
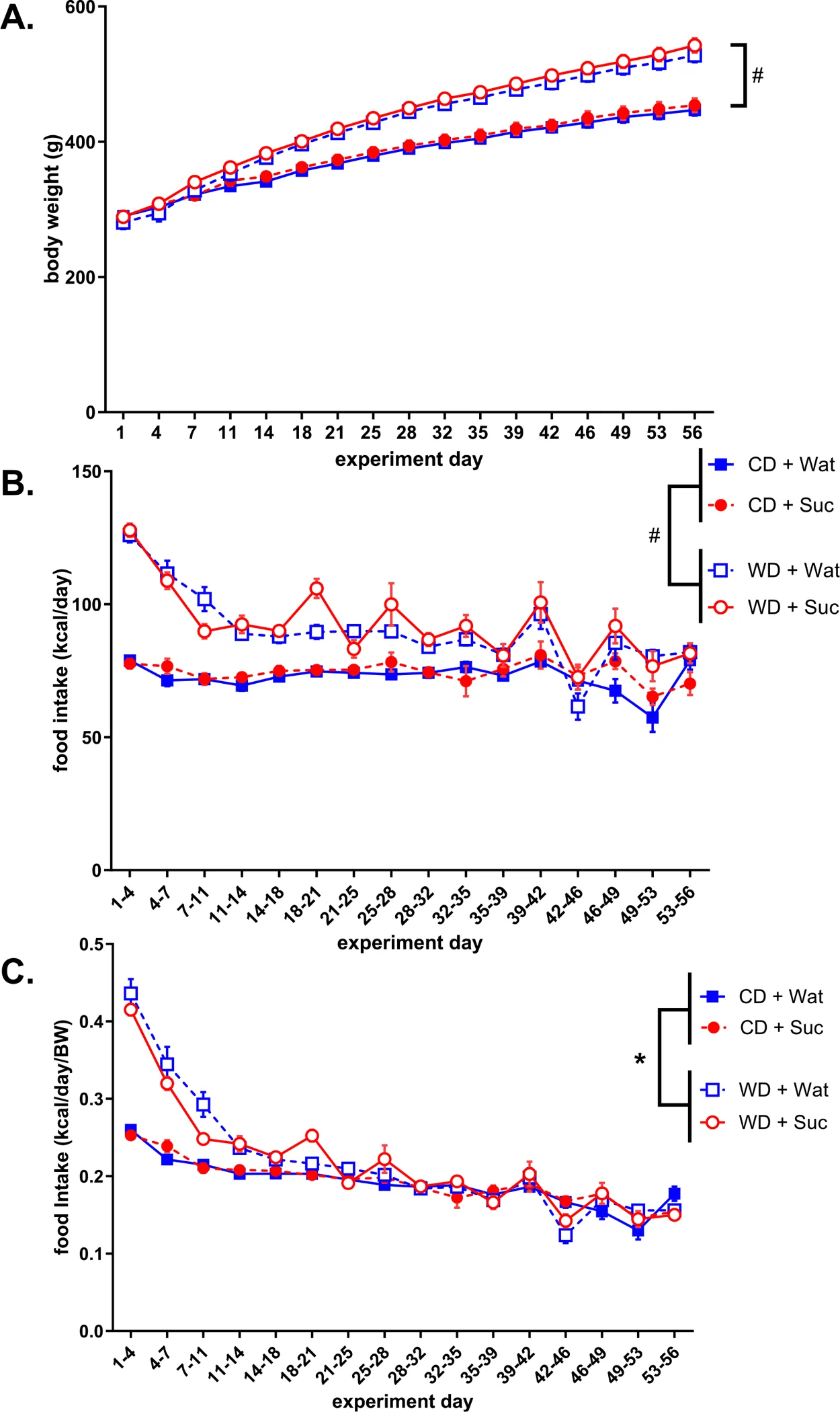
Pre-exposure to Western diet (WD; vs. normal chow diet (CD)) for 8 weeks effectively increased body weight and caloric intake prior to onset of the time-limited drink paradigm. Time course of (A) body weight, (B) caloric intake, and (C) caloric intake normalized to body weight in rats with ad libitum access to WD or CD beginning on experiment day 1. Data shown as mean ± SEM. *n* = 12 rats/group. #*p* < 0.05 indicates main effects of DIET and TIME and a DIETxTIME interaction. *<0.05 indicates main effects of DIET and TIME and interactive effects of DIETxTIME and DRINKxTIME.

Rats were given ad libitum access to WD (vs. normal chow) for 8 weeks (experiment days 1–56) to induce obesity prior to sucrose drink exposure (Fig. 1A). WD-fed rats had increased body weight compared to chow-fed rats by day 14 of diet exposure (Fig. 2A). Statistical analysis revealed main effects of DIET (F _(1, 44)_ = 44.32, *p* < 0.0001) and TIME (F _(2.023, 89.02)_ = 1953, *p* < 0.0001), and an interactive effect of DIETxTIME (F _(2.023, 89.02)_ = 92.86, *p* < 0.0001). Caloric intake in WD-fed rats was higher than chow-fed controls, particularly during the first week of diet exposure (Fig. 2B). Statistical analysis of caloric intake revealed main effects of DIET (F _(1, 44)_ = 113.7, *p* < 0.0001) and TIME (F _(7.771, 341.9)_ = 24.05, *p* < 0.0001), and an interactive effect of DIETxTIME (F _(7.771, 341.9)_ = 14.11, *p* < 0.0001). Daily caloric intake normalized to body weight was elevated at the onset of WD access but later returned to the levels of chow controls (Fig. 2C). Statistical analysis of intake normalized to body weight revealed main effects of DIET (F _(1, 44)_ = 82.39, *p* < 0.0001) and TIME (F _(7.061, 310.7)_ = 156.9, *p* < 0.0001) and an interactive effect of DIETxTIME (F _(7.061, 310.7)_ = 37.17, *p* < 0.0001). ANOVA also identified a DRINKxTIME (F _(7.061, 310.7)_ = 2.100, *p* = 0.0427) interaction, though post-hoc analyses did not identify any differences between the sucrose and water groups at any individual time points, and visually it is difficult to resolve a direction or timing for this interactive effect, indicating that it is likely of minimal magnitude.

**Fig. 3.**
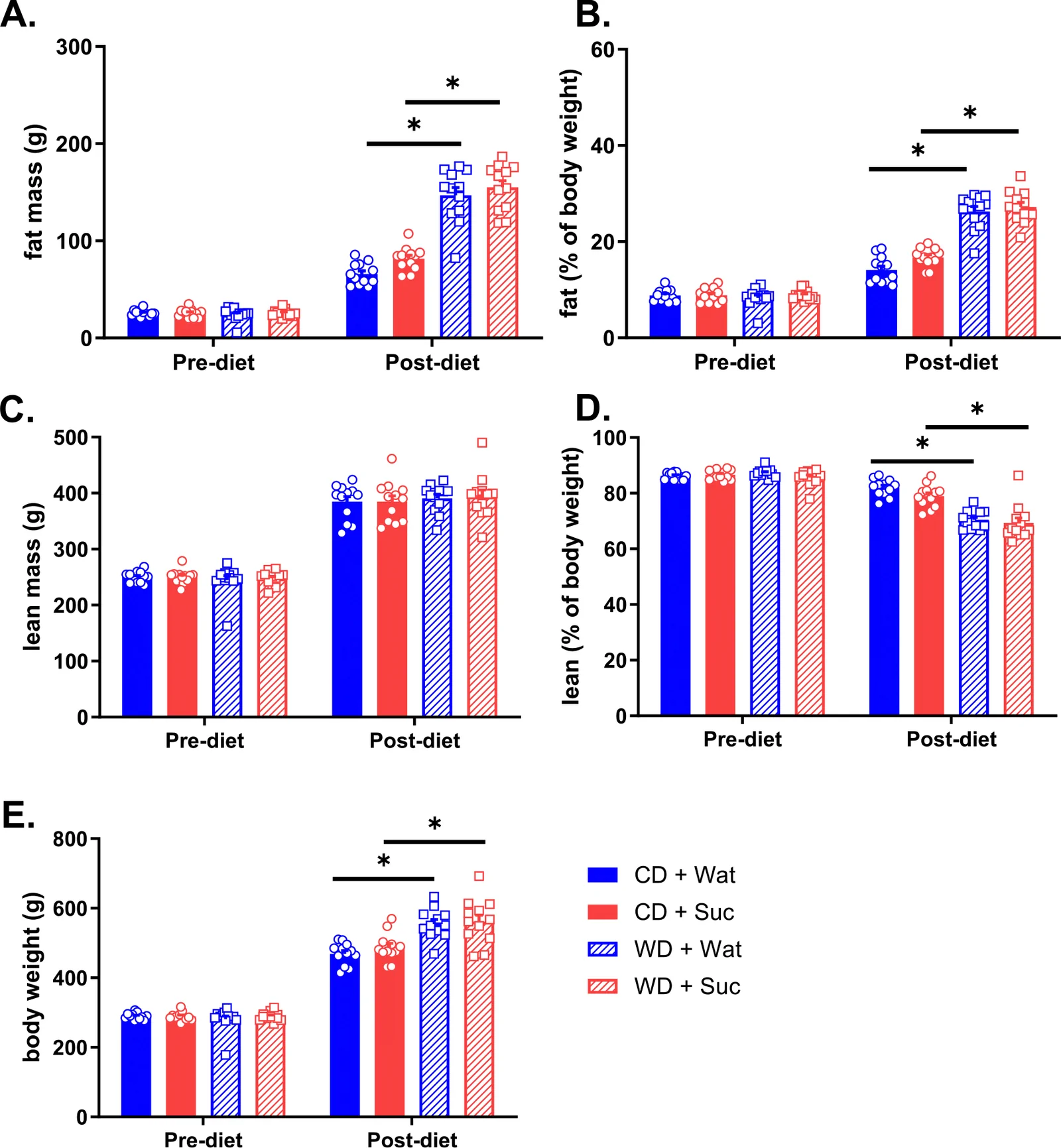
Pre-exposure to Western diet (WD; vs. normal chow diet (CD)) for 8 weeks effectively increased adiposity prior to onset of the time-limited drink paradigm. (A) Fat mass, (B) percent body fat, (C) lean mass, (D) percent lean mass, and (E) body weight on experiment days 1 (Pre-diet) and 56 (Post-diet) of ad libitum access to WD or CD. Data shown as mean ± SEM. *n* = 12 rats/group. *p < 0.05 by planned *t*-tests.

After 8 weeks of ad libitum access to WD (vs. CD controls), WD-fed rats had greater body fat mass compared to lean CD-fed rats (Fig. 3A). Statistical analysis revealed main effects of DIET (F _(1, 44)_ = 128.6, *p* < 0.0001) and TIME (F _(1, 44)_ =1209, *p* <0.0001), and an interactive effect of DIETxTIME (F _(1, 44)_ = 242.7, *p* < 0.0001). Percent body fat was also increased in WD-fed rats (Fig. 3B). Statistical analysis revealed main effects of DIET (F _(1, 44)_ = 86.04, *p* < 0.0001) and TIME F _(1, 44)_ = 1444, *p* < 0.0001), and an interactive effect of DIETxTIME (F _(1, 44)_ = 316.3, *p* < 0.0001). Of note, DRINKxTIME interactions were also identified for both fat mass (F _(1, 44)_ =4.815, *p* = 0.0335, Fig. 3A) and percent body fat (F _(1, 44)_ = 5.257, *p* = 0.0267, Fig. 3B), indicating the sucrose groups had overall greater body fat after diet exposure, though post-hoc analyses did not identify any specific differences between the sucrose vs water groups, suggesting the interactions were modest effects. During the 8 weeks of diet pre-exposure, lean mass increased to the same extent in WD-fed and CD-fed rats (Fig. 3C). Statistical analysis revealed a main effect of TIME (F _(1, 44)_ =1279, *p* <0.0001). Percent lean mass, however, was reduced in WD-fed rats relative to CD controls (Fig. 3D). Statistical analysis revealed main effects of DIET (F _(1, 44)_ = 43.48, *p* < 0.0001) and TIME (F _(1, 44)_ = 396.4, *p* < 0.0001), and an interactive effect of DIETxTIME (F _(1, 44)_ = 88.42, *p* < 0.0001). This reduction in percent lean mass resulted from a greater degree of body weight gain in WD-fed rats compared to CD-fed rats (Fig. 3E). Statistical analysis revealed a main effect of DIET (F _(1, 44)_ = 19.17, *p* < 0.0001) and TIME (F _(1, 44)_ = 1693, *p* < 0.0001), and an interactive effect of DIETxTIME (F _(1, 44)_ = 57.49, *p* < 0.0001).

Importantly, pre-exposure to WD increased body weight to the same extent for rats assigned to receive time-limited access to sucrose (Suc) vs. water (Wat), implying comparable development of Western DIO prior to beginning the drink exposure paradigm.

#### 3.1.2. Impact of time-limited sucrose (vs. water) access on drink intake, food intake, and body weight

**Fig. 4.**
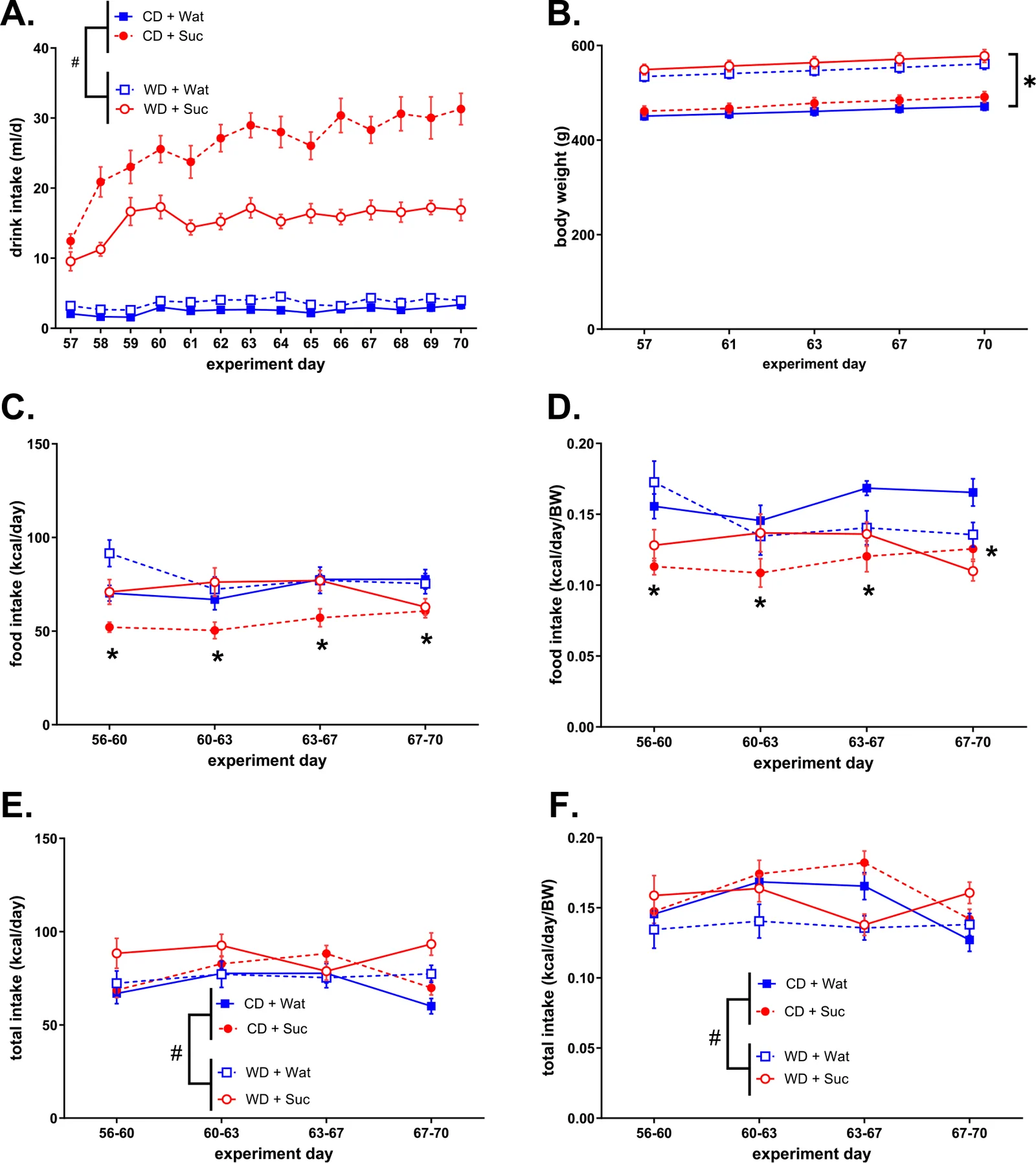
During the time-limited sucrose access paradigm on experiment days 57–70, Western diet (WD)-fed rats consumed less sucrose drink relative to chow diet (CD)-fed controls, and sucrose intake modestly increased body weight regardless of diet type. Rats were given twice daily, 30-min access to 30 % sucrose (Suc) drink vs. water (Wat) controls, in addition to ad libitum access to their respective diet and normal water bottle, for two weeks. (A) Total daily drink intake during the time-limited access periods (i.e., sum of twice-daily 30-min sessions), (B) body weight, (C) daily caloric intake from respective diet, (D) daily caloric intake from diet normalized to body weight, (E) total daily caloric intake from all sources (diet and drink), and (F) total daily caloric intake from all sources normalized to body weight. Data shown as mean ± SEM. *n* = 12 rats/group. For panel A: #*p* < 0.05 indicates main effects of DIET, DRINK, and TIME, and interactive effects of DIETxDRINK, DIETxTIME, DRINKxTIME, and DIETxDRINKxTIME. For panel B: *p < 0.05 indicates main effects of DIET and TIME and an interactive effect of DRINKxTIME. For panels C-D: *p < 0.05 for sucrose vs respective water control by planned *t*-tests. For panel E: #*p* < 0.05 indicates main effects of DIET and DRINK, and an interactive effect of DIETxTIME. For panel F: #*p* < 0.05 indicates main effects of DRINK and TIME, and an interactive effect of DIETxTIME.

On experiment days 57–70, rats remained on their respective ad libitum diet (WD vs. CD) and were additionally allowed to drink as much 30 % sucrose drink (vs. water controls) as they wanted within each 30-min session (i.e., time-limited drink access), occurring twice daily for two weeks. Rats with twice-daily time-limited access to water (as a control) drank very little regardless of diet, consistent with the lower palatability of the water (relative to 30 % sucrose) and ongoing, ad libitum access to the home cage water bottle. In contrast, rats given twice-daily time-limited access to 30 % sucrose drank a much larger volume relative to water controls (consistent with the high palatability of 30 % sucrose drink [12]), and this effect varied with diet, such that CD-fed rats consumed roughly 28 ml of sucrose solution each day, whereas WD-fed rats drank roughly 16 mL daily (Fig. 4A). Statistical analysis revealed main effects of DIET (F_(1, 44)_ = 21.28, *p* < 0.0001), DRINK (F_(1, 44)_ = 292.8, *p* < 0.0001) and TIME (F_(6.768, 297.8)_ = 21.01, *p* < 0.0001), and interactive effects of DIETxDRINK (F _(1, 44)_ = 32.66), DIETxTIME (F _(6.768, 297.8)_ = 3.844, *p* = 0.0006), DRINKxTIME (F _(6.768, 297.8)_ = 14.59, *p* < 0.0001), and DIETxDRINKxTIME (F _(6.768, 297.8)_ = 3.730, *p* = 0.0008).

Body weight remained greater in WD-fed vs. CD-fed rats during the time-limited sucrose access paradigm and was minimally affected by sucrose (vs. water) access regardless of diet type (Fig. 4B). Statistical analysis revealed main effects of DIET (F _(1, 44)_ = 62.86, *p* < 0.0001) and TIME (F _(2.721, 119.7)_ = 368.8, *p* < 0.0001), and an interactive effect of DRINKxTIME (F _(2.721, 119.7)_ = 5.399, *p* = 0.0023), indicating that sucrose drink increased body weight (relative to water controls) towards the end of the paradigm in both diet groups. However, planned *t*-test comparisons did not identify a significant impact of sucrose on body weight for either diet at any individual time point.

Consumption of the assigned ad libitum diet (WD vs. CD) was monitored throughout the time-limited access paradigm to evaluate the extent that sucrose access altered diet intake. Analysis by ANOVA indicated that daily food intake (kcal) was decreased in CD (vs. WD)-fed rats and in those with time-limited sucrose (vs. water) access (Fig. 4C; main effects of DIET (F _(1, 44)_ = 12.26, *p* = 0.0011) and DRINK (F _(1, 44)_ = 15.33, *p* = 0.0003), and an interactive effect of DIETxTIME (F _(3, 132)_ = 2.998, *p* = 0.0331)). Subsequent *t*-tests indicated that the sucrose-associated decrease in food intake occurred primarily in CD (vs. WD)-fed rats, possibly because the CD-fed rats consumed a larger amount of sucrose drink than the WD-fed rats (Fig. 4A). As WD-fed rats were overall much larger than CD controls, daily caloric intake was also analyzed after normalization to body weight (Fig. 4D). Statistical analysis revealed a main effect of DRINK (F _(1, 44)_ = 28.18, *p* < 0.0001) and interactive effects of DIETxDRINK (F _(1, 44)_ = 4.430, *p* = 0.0411) and DIETxTIME (F _(3, 132)_ = 3.059, *p* = 0.0306), indicating that sucrose access reduced dietary intake in diet-specific manner. T-test analyses further identified that sucrose reduced food intake in rats given CD, but not WD, consistent with the greater sucrose consumption of CD vs. WD rats (Fig. 4A).

The sum of calories derived from all sources (drink and diet) was calculated to evaluate the extent that sucrose access altered total caloric intake during the time-limited sucrose access paradigm. Total daily caloric intake was greater in rats fed WD (vs. CD) and was greater in those with time-limited sucrose (vs. water) access (Fig. 4E). Statistical analysis revealed main effects of DIET (F _(1, 44)_ = 5.643, *p* = 0.0219) and DRINK (F _(1, 44)_ = 8.552, *p* = 0.0054), and an interactive effect of DIETxTIME (F _(3, 132)_ = 5.465, *p* = 0.0014). Similar results were observed after normalization to body weight (Fig. 4F). Statistical analysis revealed main effects of DRINK (F _(1, 44)_ = 7.093, *p* = 0.0108) and TIME (F _(2.518, 110.8)_ = 3.676, *p* = 0.0199), and an interactive effect of DIETxTIME (F _(3, 132)_ = 6.056, *p* = 0.0007). However, planned *t*-test analyses did not identify a significant impact of either WD or sucrose on total daily caloric intake (with or without normalization to body weight) for any individual time point.

#### 3.1.3. Impact of time-limited sucrose (vs. water) access on the plasma corticosterone response to restraint stress

**Fig. 5.**
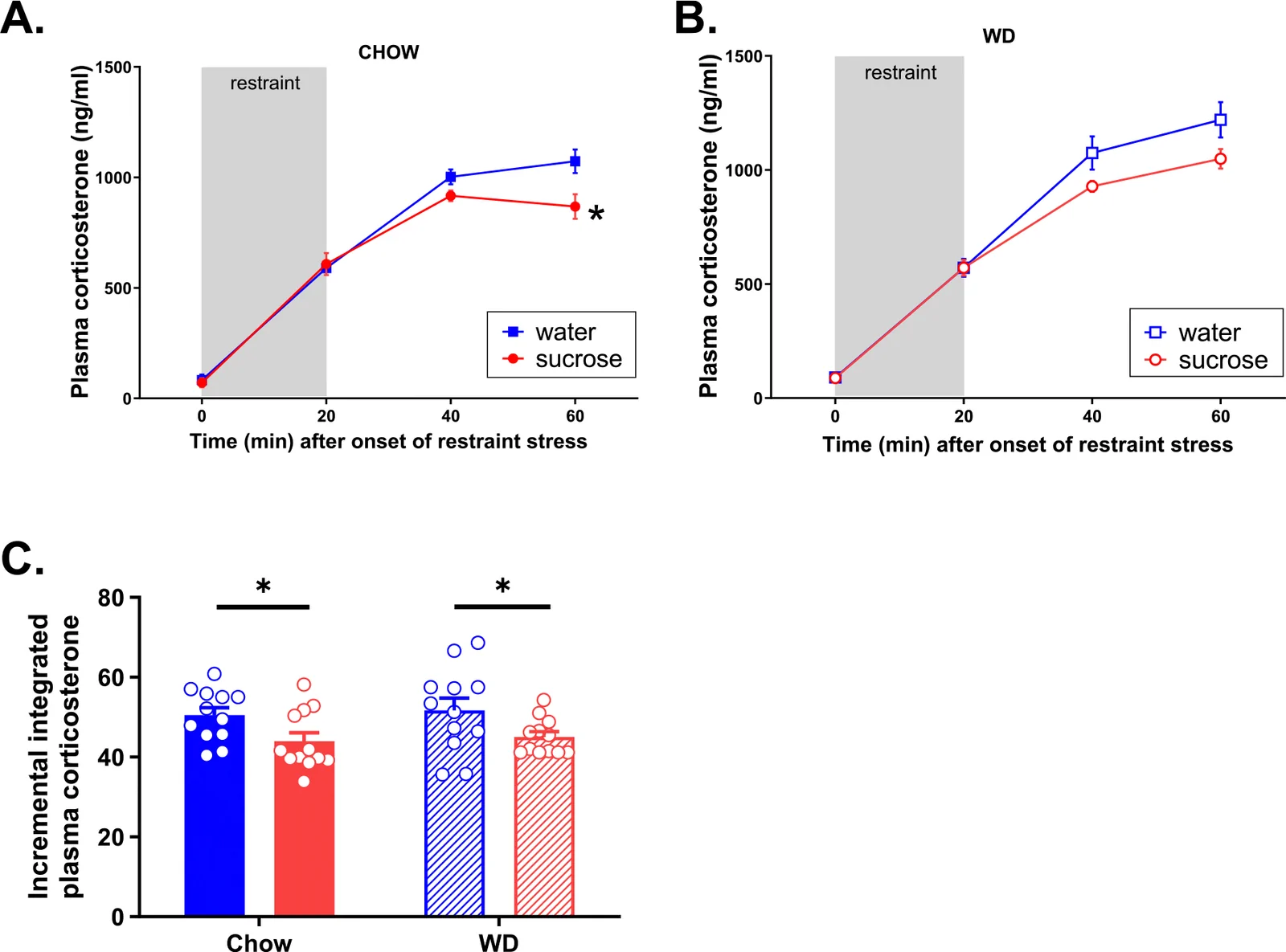
A history of time-limited sucrose access (vs. water controls) blunted the plasma corticosterone response to stress regardless of diet. The time course of the plasma corticosterone response in (A) chow diet (CD)- and (B) Western diet (WD)-fed rats, and (C) the integrated area-under-the-curve of the response. Data shown as mean ± SEM. *n* = 12 rats/group. Note that the data shown in panels A and B were analyzed together in a 3-way RM-ANOVA comparing DRINK, DIET, and TIME, and are shown separated by diet type to aid visualization. *p < 0.05 vs. water controls by planned *t*-tests.

Following two weeks of time-limited sucrose access, rats underwent an acute 20-minute restraint stressor, with collection of tail blood at several timepoints to evaluate the plasma corticosterone response. Time-limited access to sucrose drink significantly decreased plasma corticosterone levels, particularly at later post-stress timepoints regardless of diet type, whereas WD increased plasma corticosterone at later post-stress time points regardless of drink type. This is indicated by main effects of DRINK (F _(1, 44)_ =6.411, *p* =0.0150) and TIME (F _(2.536, 111.6)_ = 678.8, *p* < 0.0001), with interactive effects of DIETxTIME (F _(2.536, 111.6)_ = 6.149, *p* = 0.0013) and DRINKxTIME (F _(2.536, 111.6)_ = 7.210, *p* = 0.0004). Moreover, planned comparisons indicated that sucrose reduced plasma corticosterone at 60 min after restraint onset among CD-fed rats (Fig. 5A) and tended to decrease it at 40-min (*p* = 0.07) and 60-min (*p* = 0.07) within WD-fed rats (Fig. 5B). The incremental integrated plasma corticosterone response was calculated to evaluate the extent that diet and drink treatments impacted the cumulative plasma corticosterone response. The integrated corticosterone response was reduced in rats with prior time-limited sucrose (vs. water) access regardless of diet type (Fig. 5C). Statistical analysis revealed a main effect of DRINK (F _(1, 44)_ = 9.171 *p* = 0.0041).

### 3.2. Experiment 2: Volume-limited sucrose

#### 3.2.1. Pre-exposure to WD effectively increased caloric intake, body weight, and adiposity

**Fig. 6.**
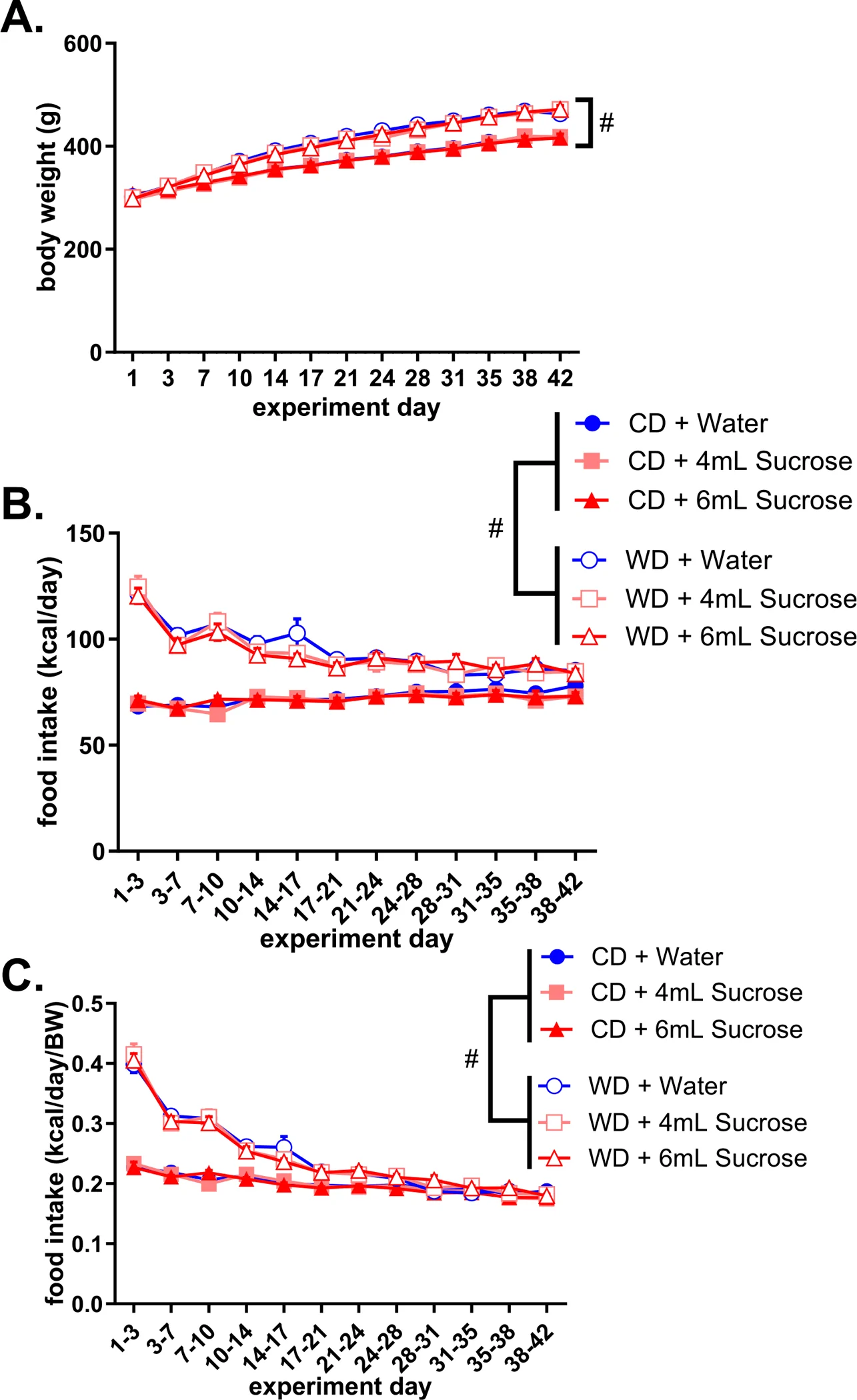
Pre-exposure to Western diet (WD; vs. normal chow diet (CD)) for 6 weeks effectively increased body weight and caloric intake prior to onset of the volume-limited drink paradigm. Time course of (A) body weight, (B) caloric intake, and (C) caloric intake normalized to body weight in rats with ad libitum access to WD or CD beginning on experiment day 1. Data shown as mean ± SEM. *n* = 12–13 rats/group. #*p* < 0.05 indicates main effects of DIET and TIME, and an interactive effect of DIETxTIME.

Rats were given ad libitum access to WD (vs. normal chow) for 6 weeks (experiment days 1–42) to induce obesity prior to sucrose drink exposure (Fig. 1B). WD-fed rats had increased body weight compared to CD-fed rats (Fig. 6A). Statistical analysis revealed main effects of DIET (F_(1,69)_=58.3844, *p* < 0.001) and TIME (F_(2.90,199.81)_=1357.287, *p* < 0.001), and an interactive effect of DIETxTIME (F_(2.90,199.81)_=50.872, *p* < 0.001). Caloric intake in WD-fed rats was higher than chow-fed controls throughout diet exposure, particularly during the first week of diet exposure (Fig. 6B). Statistical analysis revealed main effects of DIET (F_(1,69)_=210.52, *p* < 0.001) and TIME (F_(5.77,397.95)_=31.016, *p* < 0.001), with an interactive effect of DIETxTIME (F_(5.77,397.95)_=57.635, *p* <0.001). Daily caloric intake normalized to body weight was elevated in WD-fed rats at the onset of diet exposure but later returned to the levels of CD-fed controls (Fig. 6C). Statistical analysis revealed main effects of DIET (F_(1,69)_=267.006, *p* < 0.001) and TIME (F_(4.99,344.12)_=273.052, *p* < 0.001), and an interactive effect of DIETxTIME (F_(4.99,344.12)_=114.773, *p* < 0.001).

**Fig. 7.**
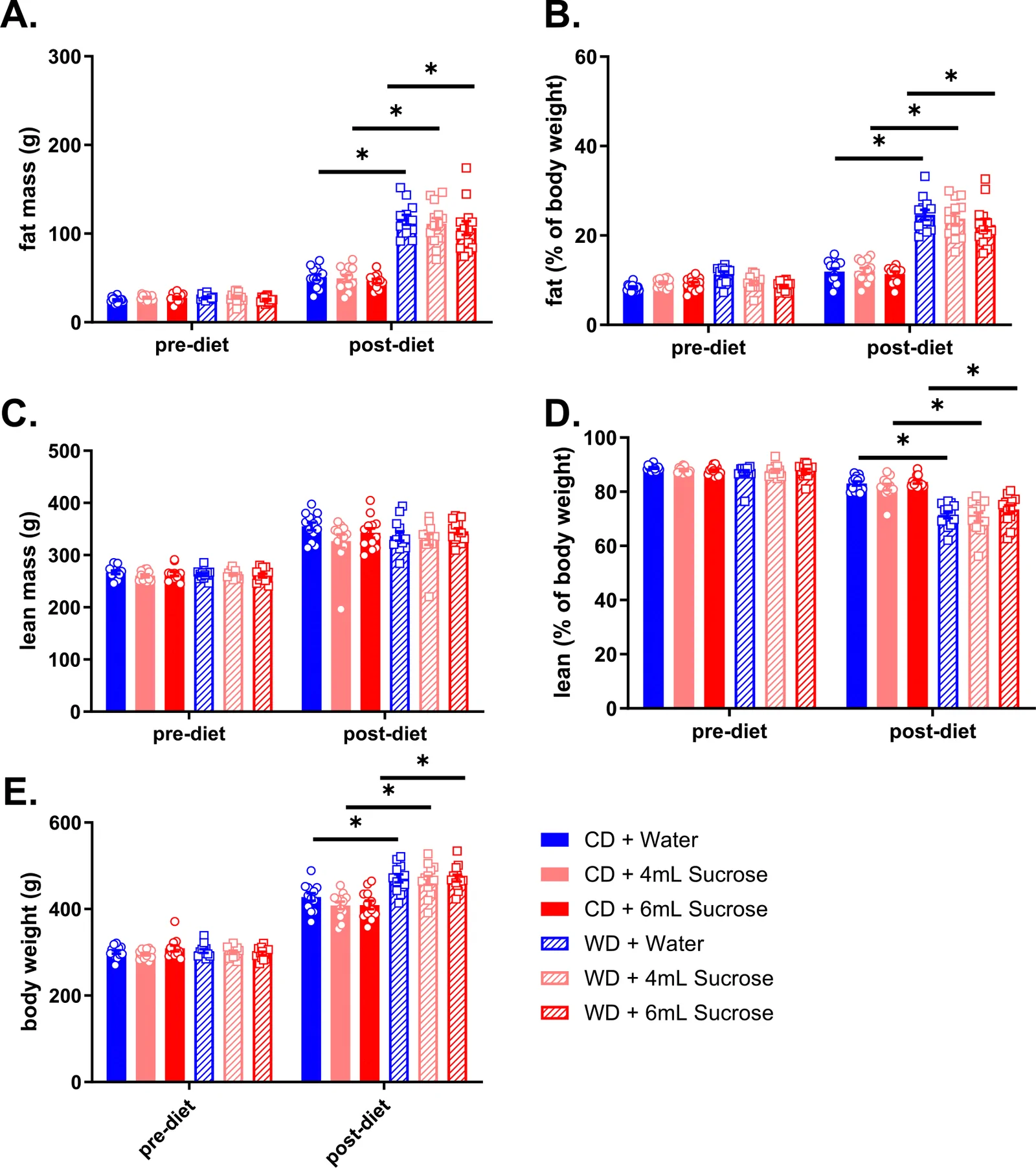
Pre-exposure to Western diet (WD; vs. normal chow diet (CD)) for 6 weeks effectively increased adiposity prior to onset of the volume-limited drink paradigm. A) Fat mass, (B) percent body fat, (C) lean mass, (D) percent lean mass, and (E) body weight on experiment days 1 (Pre-diet) and 42 (Post-diet) of ad libitum access to WD or CD. Data shown as mean ± SEM. *n* = 12–13 rats/group. *p < 0.05 by planned *t*-tests.

After 6-weeks of ad libitum access to WD (vs. CD controls), WD-fed rats had more body fat mass than lean CD-fed controls (Fig. 7A). Statistical analysis revealed main effects of DIET (F_(1,69)_=170.928, *p* < 0.001) and TIME (F_(1,69)_=695.250, *p* < 0.001), and an interactive effect of DIETxTIME (F_(1,69)_=234.436, *p* < 0.001). Percent body fat was also increased in WD-fed rats (Fig. 7B). Statistical analysis revealed main effects of DIET (F_(1,69)_=170.18, *p* < 0.001) and TIME (F_(1,69)_=480.467, *p* < 0.001), and an interactive effect of DIETxTIME (F_(1,69)_=210.814, *p* < 0.001). During the 6-weeks of diet pre-exposure, lean mass was increased to the same extent in WD-fed and CD-fed rats (Fig. 7C). Statistical analysis revealed a main effect of TIME (F_(1,69)_=453.547, *p* < 0.001). However, percent lean mass was reduced in WD-fed rats in comparison to CD-fed controls (Fig. 7D). Statistical analysis revealed main effects of DIET (F_(1,69)_=59.328, *p* < 0.001) and TIME (F_(1,69)_=298.6003, *p* < 0.001), and an interactive effect of DIETxTIME (F_(1,69)_=58.9104, *p* < 0.001). This reduction in percent lean mass was due to a greater increase in body weight in WD-fed rats relative to CD-fed controls (Fig. 7E). Statistical analysis revealed main effects of DIET (F_(1,69)_=34.074, *p* < 0.001) and TIME (F_(1,69)_=1523.166, *p* < 0.001), and an interactive effect of DIETxTIME (F_(1,69)_=59.860, *p* < 0.001).

Importantly, pre-exposure to WD increased body weight, caloric intake, and adiposity to the same extent for rats assigned to receive volume-limited access to sucrose (4 mL sucrose, 6 mL sucrose) vs. water (4 mL water), confirming comparable development of Western DIO prior to onset of the drink exposure paradigm.

#### 3.2.2. Impact of volume-limited sucrose (vs. water) access on drink intake, food intake, and body weight

**Fig. 8.**
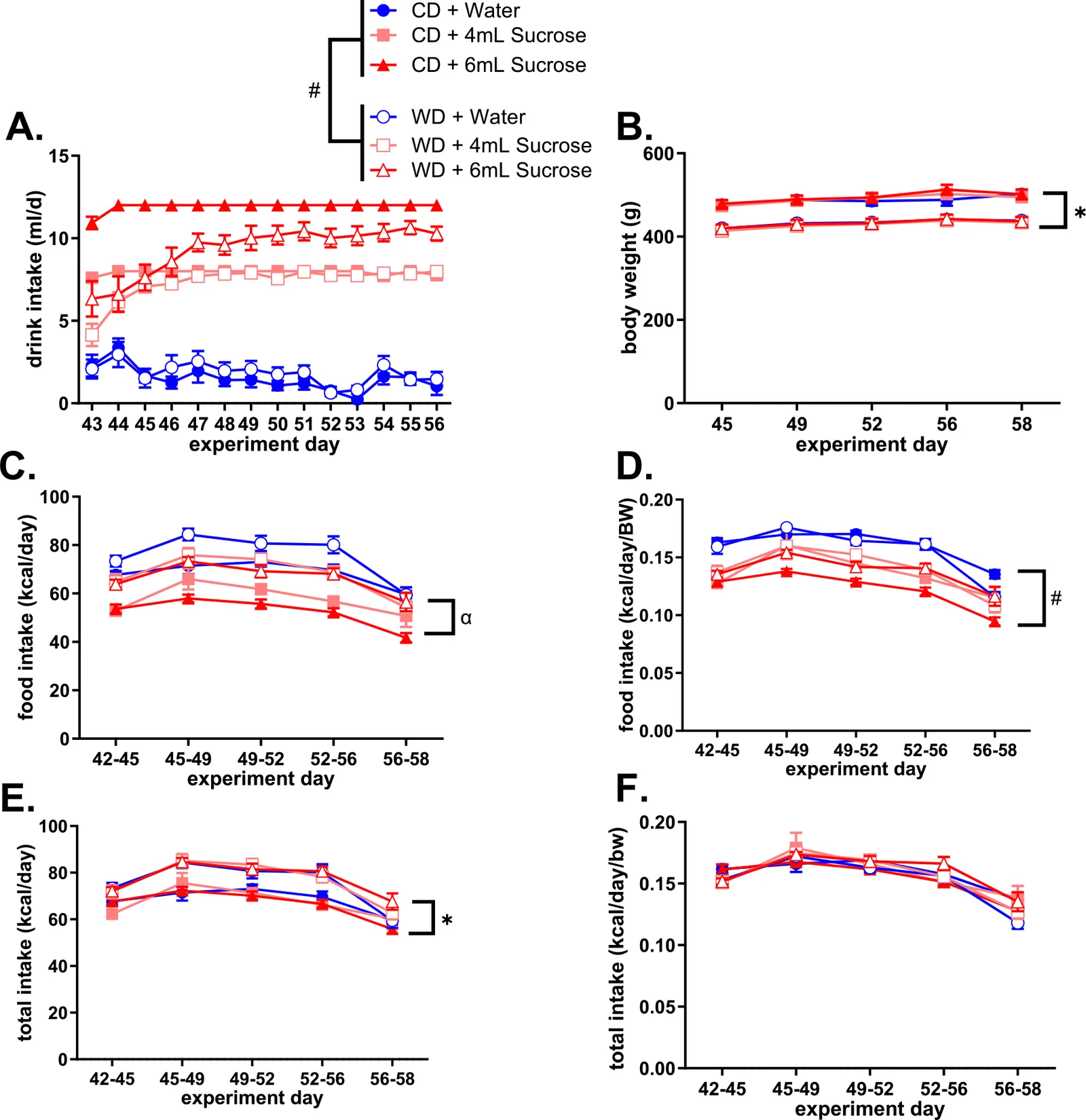
During the volume-limited sucrose access paradigm on experiment days 43–57, both Western diet (WD)-fed rats and chow diet (CD)-fed controls consumed the sucrose drink and reduced their diet intake to compensate for the sucrose calories. Rats were given twice daily access to 4 mL of 30 % sucrose, 6 mL of 30 % sucrose, or 4 mL water controls in addition to ad libitum access to their respective diet and normal water bottle, for two weeks. (A) Total daily drink intake during the volume-limited access periods (i.e., sum of twice-daily sessions), (B) body weight, (C) daily caloric intake from respective diet, (D) daily caloric intake from diet normalized to body weight, (E) total daily caloric intake from all sources (diet and drink), and (F) total daily caloric intake from all sources normalized to body weight. Data shown as mean ± SEM. *n* = 12–13 rats/group. For panel A: #*p* < 0.05 indicates main effects of DIET, DRINK, and TIME, and interactive effects of DIETxDRINK, DIETxTIME, DRINKxTIME, and DIETxDRINKxTIME. For panel B: *p < 0.05 indicates main effects of DIET and TIME. For panel C: αp<0.05 indicates main effects of DIET, DRINK, and TIME, and an interactive effect of DIETxTIME. For panel D: #*p* < 0.05 indicates main effects DRINK and TIME, and an interactive effect of DRINKxDIET. For panel E: *p < 0.05 indicates main effects of DIET and TIME, and an interactive effect of DIETxTIME. Not shown on panel F: there is a main effect of TIME.

On experiment days 43–57, rats remained on their respective ad libitum diet (WD vs. CD) and were additionally allowed to drink up to 4 mL or 6 mL of 30 % sucrose drink (vs water control), occurring twice daily for two weeks. Rats with twice-daily volume-limited access to water (as a control) drank very little, regardless of diet, consistent with the lower palatability of the water (relative to 30 % sucrose) and ongoing, ad libitum access to the home cage water bottle. Rats given twice-daily access to 4 mL of 30 % sucrose drink the maximum allowed (8 ml/day) regardless of diet (Fig. 8A). Among rats offered twice-daily access to 6 ml sucrose drink, the chow-fed rats consumed the maximum volume allowed (12 ml/day), whereas the Western DIO rats consumed nearly the full amount allowed (~10 ml/day). Statistical analysis revealed main effects of DIET (F_(1,69)_=17.7, *p* < 0.001), DRINK (F_(2,69)_=540.2, *p* < 0.001) and TIME (F_(5.83,402.37)_=10.20, *p* < 0.001), and interactive effects of DIETxDRINK (F_(2,69)_=13.7, *p* < 0.001), DIETxTIME (F_(5.83,402.37)_=9.34, *p* < 0.001), DRINKxTIME (F_(11.66,402.37)_=9.58, *p* < 0.001), and DIETxDRINKxTIME (F_(11.66,402.37)_=2.17, *p* = 0.013).

Body weight remained greater in WD-fed rats vs. CD-fed rats during the volume-limited sucrose paradigm and was not affected by sucrose vs. water regardless of diet type (Fig. 8B). Statistical analysis revealed main effects of DIET (F_(1,69)_=59.443, *p* < 0.001) and TIME (F_(2.08,143.76)_=59.793, *p* < 0.001).

Consumption of the assigned ad libitum diet was monitored throughout the volume-limited sucrose paradigm to evaluate the extent that sucrose access altered diet intake. Daily food intake (kcal) was greater in rats fed WD (vs. CD) and reduced in those given access to sucrose (vs. water) (Fig. 8C). Statistical analysis revealed main effects of DIET (F_(1,69)_=42.35, *p* < 0.001), DRINK (F_(2,69)_=22.62, *p* < 0.001), and TIME (F_(2.79,192.78)_=73.04, *p* < 0.001), and an interactive effect of DIETxTIME (F_(2.79,192.78)_=3.09, *p* = 0.031). Food intake was normalized to body weight (Fig. 8D), as WD-fed rats were much larger than CD-controls. Statistical analysis revealed main effects of DRINK (F_(2,69)_=45.41, *p* < 0.001) and TIME (F_(3.05,210.22)_=83.99, *p* < 0.001), and an interactive effect of DRINKxDIET (F_(2,69)_=5.64, *p* = 0.005), indicating that after normalization to body weight, 6 mL sucrose access reduced diet intake to a greater extent in CD-fed (vs. WD-fed) rats, likely because the WD rats did not drink the full amount of offered sucrose (Fig. 8A).

Total intake from all sources (diet and drink) was calculated to evaluate the extent that sucrose access altered total intake during the volume-limited sucrose paradigm. Total daily caloric intake was greater in rats fed WD (vs. CD) and not impacted by drink type (Fig. 8E). Statistical analysis revealed main effects of DIET (F_(1,69)_=34.246, *p* < 0.001) and TIME (F_(2.79,192.80)_=81.11, *p* < 0.001) and an interactive effect of DIETxTIME (F_(2.79,192.80)_=4.35, *p* = 0.007). After normalization to body weight (Fig. 8F), caloric intake was not impacted by either diet or drink types, as statistical analysis revealed only a main effect of TIME (F_(2.93,202.02)_=77.41, *p* < 0.001).

#### 3.2.3. Impact of volume-limited sucrose (vs. water) access on the plasma corticosterone response to restraint stress

**Fig. 9.**
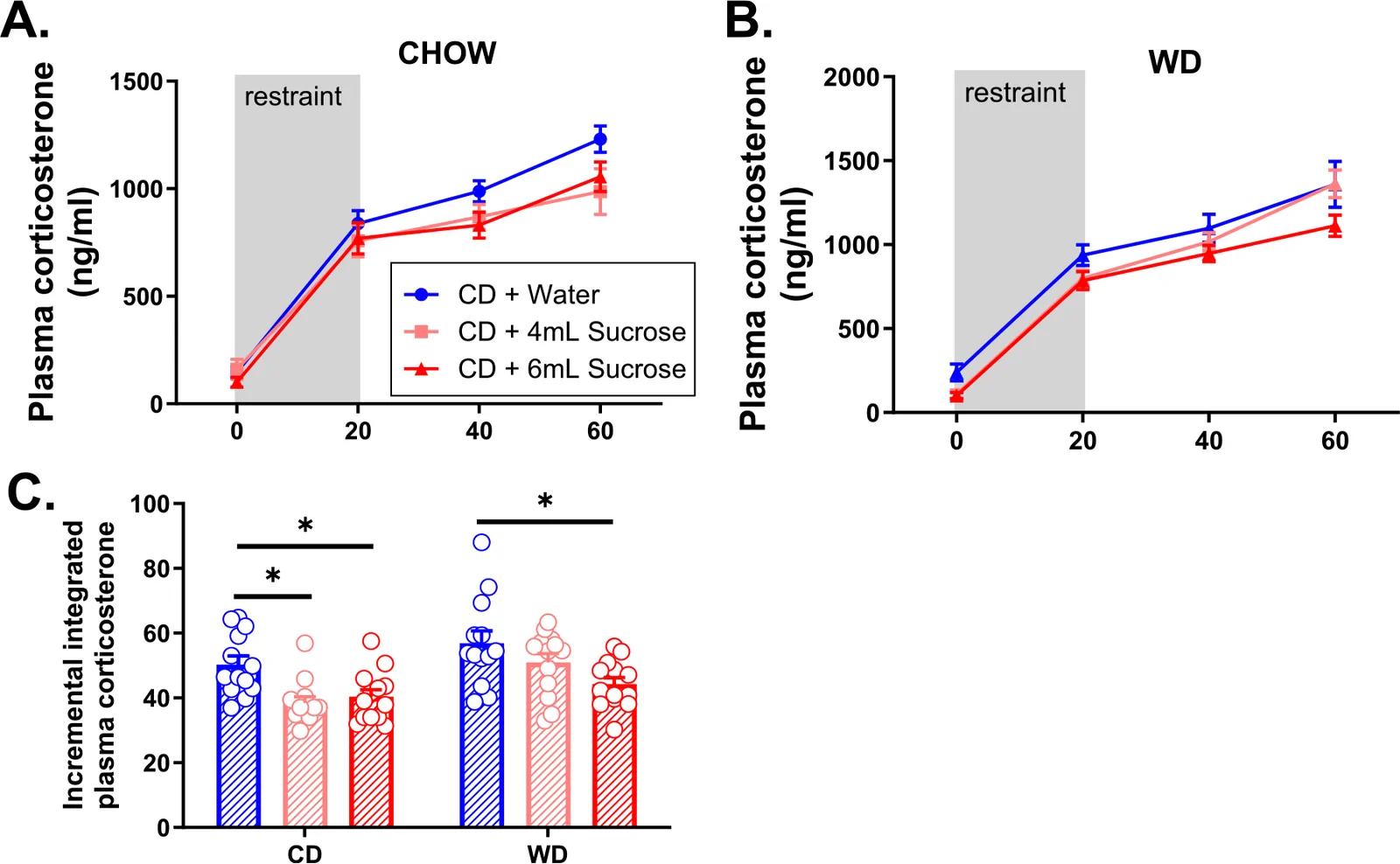
A history of volume-limited sucrose access (vs. water controls) blunted the plasma corticosterone response to stress in a diet-dependent manner. The time course of the plasma corticosterone response in (A) chow diet (CD)- and (B) Western diet (WD)-fed rats, and (C) the integrated area-under-the-curve of the response. Data shown as mean ± SEM. *n* = 12–13 rats/group. Note that the data shown in panels A and B were analyzed together in a 3-way RM-ANOVA comparing DRINK, DIET, and TIME, and are shown separated by diet type to aid visualization. *p < 0.05 vs. water controls by planned *t*-tests.

Following two-week volume-limited sucrose access, rats underwent an acute 20-minute restraint stressor to measure the plasma corticosterone responses. Statistical analysis of the plasma corticosterone time course (Fig. 9A,B) revealed main effects of DIET (F_(1,62)_=6.630, *p* = 0.012), DRINK (F_(2,62)_=5.079, *p* = 0.009), and TIME (F_(2.05,127.23)_=452.550, *p* < 0.001), and an interactive effect of DIETxTIME (F_(2.05,127.23)_=3.448, *p* = 0.034), indicating the sucrose (vs. water) drink reduced post-stress plasma corticosterone, whereas WD (vs. CD) increased it. The incremental integrated plasma corticosterone response was calculated to evaluate the extent that sucrose access impacted the cumulative plasma corticosterone response. The integrated corticosterone response was reduced in rats with prior sucrose (vs. water) access regardless of diet type or volume. Statistical analysis revealed main effects of DIET (F _(1,72)_ =12.66, *p* =0.0007) and DRINK (F _(2, 72)_ = 10.19, *p* = 0.0001), with planned *t*-tests indicating reduced plasma corticosterone by both 4 and 6 ml/session in CD-fed rats, and by 6 ml/session in WD-fed rats.

## 4. Discussion

### 4.1. Summary

The present work tested the hypothesis that greater amounts of sucrose intake are needed to confer effective HPA axis-blunting in the context of dietary obesity. Two different behavioral approaches (time-limited and volume-limited sucrose drink access) were used to allow a larger amount of sucrose consumption than previously studied, and the effects on HPA axis stress responses were compared between lean vs. DIO rats. Time-limited sucrose access (twice daily for 30 min) resulted in much greater sucrose drink intake (~16ml/d) than that allowed in the LSI paradigm (4 ml/twice-daily session, or 8 ml/d) and reduced post-stress plasma corticosterone relative to water controls in both lean and DIO rats. However, the DIO rats drank less sucrose than chow-fed lean controls during the time-limited access paradigm, so we also utilized a complementary approach – providing twice daily volume-limited access to a larger sucrose volume (6 ml vs. the standard 4 ml per twice-daily session). Planned comparisons indicated that volume-limited access to 6 ml sucrose drink twice-daily reduced the plasma corticosterone response to restraint stress in both lean and DIO rats, whereas the 4 ml volume was only effective in lean rats. These data replicate our prior finding that the typical LSI sucrose volume (4 ml/session) does not reduce the HPA axis response to stress during Western DIO [11], and extend this to show that larger sucrose volumes, given via either time- or volume-limited access paradigms, recover this effect.

### 4.2. Impact of Western DIO on time- vs. volume-limited sucrose drink intake, body weight, and total caloric intake

Prolonged feeding with a WD for 8 weeks (experiment 1) or 6 weeks (experiment 2) effectively induced dietary obesity – characterized by an increase in body weight, adiposity, and overall calories consumed – prior to beginning the drink access paradigm. As previously seen with the typical LSI paradigm [11,13,15], rats with volume-limited access (experiment 2) to either 8 ml/d or 12 ml/d of sucrose drink (divided into two daily session) reduced the intake of their assigned diet in a manner proportional to the amount of sucrose consumed. This resulted in similar total caloric intake and body weight among the drink groups. However, rats with time-limited access (experiment 1) to sucrose drank much larger volumes (~28 ml/d and ~16 ml/d in CD- and WD-fed rats, respectively) than those with volume-limited access. Rats with time-limited sucrose access again reduced the intake of their assigned diet in a manner proportional to the amount of sucrose consumed, though this decrease did not fully compensate the calories provided by the sucrose, such that total caloric intake was greater in rats with sucrose access and body weight was slightly higher in these rats by the end of the experiment. These data suggest that rats are less able to compensate for the calories provided by the sucrose (i.e., by reducing their diet intake) as larger amounts of sucrose are consumed.

When larger volumes of sucrose drink (i.e., ≥ 6 ml/session) were offered to WD-fed rats, they chose to drink less of it than CD-fed controls, regardless of the type of access paradigm. This effect was most pronounced during the time-limited paradigm, during which WD-fed rats drank ~40 % less than the CD-fed rats, though it also occurred to a more modest extent (~15 % less) during volume-limited access. These results are similar to prior findings that rats with Western DIO consume less sucrose when it is freely offered [26]. The reasons for this are not known but may reflect combined effects of the WD itself, and/or its associated effects on body weight/adiposity and metabolic dysfunction. Sucrose may be more preferred when it is offered in the context of (or in contrast to) a low palatability chow diet, as opposed to a high palatability WD. In support of this idea, when DIO mice are switched from a WD to a standard chow diet for 10 days, sucrose consumption – which was previously reduced in DIO vs lean mice – increased to the level of lean controls despite continuing obesity [26]. Likewise, switching dietary obese mice to standard chow increased progressive ratio responding for sucrose within 3 days of WD withdrawal [27]. These findings suggest that the consumption of a highly palatable (e.g., high fat) diet can decrease the value of other palatable foods, and this can be reversed by short term placement onto a low palatability chow diet in the absence of any body weight loss [26,28]. However, obesity is also considered a state of consummatory reward deficit, such that DIO rats and humans with obesity have less activation of brain reward regions in response to eating palatable foods [20,29,30]. Indeed, there seems to be a negative correlation between adiposity and the hedonic response to sucrose; as adiposity increases, sucrose consumption decreases [31]. Formerly obese rats that have undergone weight loss through caloric restriction of WD show increased sucrose preference and consume sucrose to a similar degree as never obese lean controls [31–33], implicating the adverse metabolic consequences of WD, as opposed to WD per se, in decreasing sucrose intake. This idea is further supported by findings that neither short nor long-term WD exposure reduces sucrose consumption in the absence of elevated body weight. When lean mice were given access to a WD for 48 hrs – a duration that does not increase body weight – sucrose consumption was increased compared to DIO mice [26]. Similarly, mice that are pair-fed WD (to match the caloric intake of low-fat diet controls) have greater sucrose preference than ad libitum WD-fed DIO mice [34]. Taken together, these findings indicate that both the WD itself, as well as its negative metabolic effects (e.g., obesity), are likely to contribute to the reduced sucrose preference and consumption that is observed in the context of DIO.

This idea has important implications for the stress-blunting effects of sucrose drink, as the hedonic and rewarding properties of sucrose drink are necessary for reduced HPA axis stress responsivity [17,18]. It suggests a scenario whereby both the WD and its associated metabolic dysfunction act to reduce sucrose preference, thereby limiting its ability to blunt stress responses. Future studies can directly test this idea, for example by using short-term WD exposure or long-term WD pair-feeding to test the impact of WD in the context of limited body weight gain, and/or switching WD-fed DIO rats to standard chow to identify the extent that recovery of sucrose’s HPA blunting occurs prior to (vs. concordant with) subsequent weight loss. Though it is important to note that some of these dietary manipulations could introduce new confounds to interpretation as they may themselves alter HPA responsivity. For example, switching rats from highly palatable diets to standard chow has been shown to increase HPA axis activation, and pair-feeding is a form of food restriction, which could alter HPA axis activation and act as a stressor [35–37].

### 4.3. Larger volumes of sucrose drink intake restore effective HPA axis blunting in DIO rats

The effects of both time-limited and volume-limited sucrose access were compared to examine the relationship between the volume of 30 % sucrose consumed and the plasma corticosterone response to restraint stress. Among CD-fed lean rats, all volumes of sucrose drink, even as low as 8 ml/d (divided into 2 daily sessions, were sufficient to decrease post-stress plasma corticosterone, consistent with prior reports using the standard LSI drink paradigm [12,14,16]. In contrast, this low sucrose drink volume did not affect the post-stress plasma corticosterone among WD-fed rats with dietary obesity, as previously observed [11]. However, allowing WD-fed rats to drink a greater volume of sucrose, through either a time- or volume-limited access paradigm, recovered the HPA axis blunting. This suggests that the extent of the sucrose-induced HPA blunting depends on reaching a threshold volume of sucrose drink intake, and that this threshold is higher in rats with Western DIO. Moreover, the fact that modest increases in sucrose intake (e.g., from 8 ml/d to ~10 ml/d in experiment 2) are sufficient to restore HPA blunting in Western DIO rats has two important implications. First, it supports the idea that individuals with obesity could readily increase their palatable food intake by the amount needed to maintain effective stress relief. Second, it suggests that factors beyond sucrose volume may also contribute. For example, WD-fed rats offered the larger volumes of sucrose were allowed to drink to satiety, as evidenced by the observation by lab personnel that WD-fed rats ceased drinking sucrose well before the 30-min time limit in experiment 1, and the fact that the WD-fed rats chose to stop drinking prior to consuming the full volume of sucrose offered in the 6ml/session groups (experiment 2). This suggests that during dietary obesity, reduced HPA stress responses following a history of voluntary palatable food consumption may depend on both the amount of food consumed, as well as the degree of satiation that is reached. Future experiments will be needed to evaluate this possibility. Previous work in lean rats found LSI blunted HPA axis responses to multiple types of stress, such as restraint and hypoxia, and reduced anxiety-related behaviors [15,38,39]. While the current experiments focused on LSI’s effect on the HPA axis response to restraint stress, we expect that these findings would generalize to other stressors, such that dietary obesity would also limit LSI-blunting of other stress responses and that these effects would be recovered by larger amounts of sucrose drink. Future studies will be needed to confirm this effect.

These experiments focused on the impact of dietary obesity and increased sucrose intake on HPA stress responsivity in male rats. However, many studies suggest that women may be more prone to “comfort” feeding than men, a behavior that fluctuates across the menstrual cycle [6,40–42]. Further, both comfort feeding and stress levels are strongly associated with obesity in women [40,43]. Our previous work in lean rats has explored sex differences in food-mediated stress relief, finding that LSI reduces the HPA axis response to acute stress and reduces anxiety-like behaviors in females primarily during proestrus/estrus [15, 16]. Future work will test the impact of DIO on LSI stress-blunting in females to determine the extent that dietary obesity exerts sex-and/or estrous cycle-dependent effects.

### 4.4. Perspectives

We have previously shown that a history of limited palatable food intake consistently reduces the HPA axis response to stress, and that this occurs with a variety of food types, including sucrose, saccharin (a non-caloric sweetener) and cheese (a high-fat, low-carbohydrate food)[17, 18]. The HPA axis blunting also occurs after intermittent access to another type of natural reward (sexual activity) and is prevented when the rewarding properties of sucrose are minimized by direct gavage into the stomach [17]. This suggests that the natural reward provided by the LSI paradigm is necessary for subsequent stress relief. When taken together with the present results, the findings support the idea that obesity is a state of consummatory reward deficit, such that larger amounts of palatable food are needed to maintain effect HPA-blunting. Indeed, not only are individuals with obesity or overweight more likely to engage in stress eating [6,7,44], but stress also increases in palatable food consumption to a greater extent in those that are already overweight or obese [29,45,46]. This suggests a cycle in which one must continually increase palatable food consumption to obtain effective stress relief in response to worsening dietary obesity.
